# Crystallization of a nonreplicating rotavirus vaccine candidate

**DOI:** 10.1002/bit.27699

**Published:** 2021-02-19

**Authors:** Moo Sun Hong, Kawaljit Kaur, Nishant Sawant, Sangeeta B. Joshi, David B. Volkin, Richard D. Braatz

**Affiliations:** ^1^ Department of Chemical Engineering Massachusetts Institute of Technology Cambridge Massachusetts USA; ^2^ Department of Pharmaceutical Chemistry, Vaccine Analytics and Formulation Center University of Kansas Lawrence Kanas USA

**Keywords:** crystallization modeling, protein crystallization, rotavirus, vaccine development

## Abstract

Nonreplicating rotavirus vaccine (NRRV) candidates are being developed with the aim of serving the needs of developing countries. A significant proportion of the cost of manufacturing such vaccines is the purification in multiple chromatography steps. Crystallization has the potential to reduce purification costs and provide new product storage modality, improved operational flexibility, and reduced facility footprints. This communication describes a systematic approach for the design of the crystallization of an NRRV candidate, VP8 subunit proteins fused to the P2 epitope of tetanus toxin, using first‐principles models and preliminary experimental data. The first‐principles models are applied to literature data to obtain feasible crystallization conditions and lower bounds for nucleation and growth rates. Crystallization is then performed in a hanging‐drop vapor diffusion system, resulting in the nucleation and growth of NRRV crystals. The crystals obtained in a scaled‐up evaporative crystallization contain proteins truncated in the P2 region, but have no significant differences with the original samples in terms of antibody binding and overall conformational stability. These results demonstrate the promise of evaporative crystallization of the NRRV.

## Graphical abstract

The crystallization of an nonreplicating rotavirus vaccine (NRRV) candidate was designed by a systematic approach using first‐principles models and preliminary experimental data. The first‐principles models are applied to literature data to obtain feasible crystallization conditions and lower bounds for nucleation and growth rates. Proof‐of‐concept experiments resulted in the nucleation and growth of NRRV crystals which have no significant differences with the original samples in terms of antibody binding and overall conformational stability.

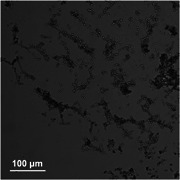

## 1 INTRODUCTION

Rotaviruses are one of the main causes of severe gastroenteritis among children, causing ~200,000 annual mortalities in less than 5 years of age worldwide with more than 90% occurring in low‐income and low‐middle‐income countries (Tate et al., 2016). To alleviate the issues of efficacy and cost effectiveness with live attenuated rotavirus vaccines, several nonreplicating rotavirus vaccine (NRRV) candidates are being developed. The most advanced of the candidates are truncated VP8 subunit proteins fused to the P2 epitope of tetanus toxin (Kirkwood et al., 2019). These NRRV antigens are currently expressed intracellularly in *Escherichia coli* and purified through a multistep process including three chromatography steps (Fix et al., 2015).

Because of its high resolution, chromatography is the most widely used separation method in bioprocesses. On the other hand, crystallization has proved to be an inexpensive industrial separation method for inorganic and organic molecules for satisfying adequate purity and production. In contrast to chromatography whose operating costs scale linearly with throughput, the operating costs for crystallization scale sublinearly (Hong et al., 2018). Although the purification of some therapeutic proteins such as insulin have used crystallization, crystallization technology effective for large‐molecule therapeutic proteins is still lacking and needs to be developed.

Technology for the design and control of the crystallization of proteins is much less mature than for small molecules. Methods for inducing supersaturation are also more limited due to the need of maintaining protein stability and quality. Many proteins are easily denatured by changes in temperature and pH, addition of precipitants, and agitation. Proteins have complex thermodynamics, slow kinetics, large uncertainties, and potential for protein aggregation that greatly restrict allowable paths through the phase diagram, which is equivalent to threading an unknown narrow winding path through an uncertain high‐dimensional space.

This communication describes a systematic approach to the design of the NRRV crystallization by a combination of first‐principles models and preliminary experimental data. Literature‐reported results for truncated VP8 subunit proteins of rotaviruses (Dormitzer et al., 2002; Kraschnefski et al., 2008, 2005; Scott et al., 2005; Yu et al., 2008; Zhang et al., 2007) are analyzed to obtain feasible crystallization conditions and lower bounds on the crystal nucleation and growth rates. Proof‐of‐concept crystallization experiments are performed for validation of the analysis and characterization of the crystals.

## 2 RESULTS AND DISCUSSION

### 2.1 Estimation of crystallization rates

Preliminary well‐ or vial‐based experimental data can provide lower bounds on crystallization rates. Most experimental studies apply screening methods such as hanging‐ or sitting‐drop vapor diffusion systems (McPherson, 2004). Vapor diffusion systems place a droplet containing protein, buffer, and precipitant in vapor equilibrium with a reservoir containing higher concentration of buffer and precipitant. Water evaporates, which increases the concentration of protein and precipitant, until the droplet reaches equilibrium with the reservoir. This process produces a gradual increase of supersaturation, resulting in nucleation and growth of crystals.

Nucleation within such drops is describable by the stochastic model (Goh et al., 2010):

(1) $$\frac{dP_{0}\left(t\right)}{dt}=-B_{0}\left(t\right)V\left(t\right)P_{0}\left(t\right),\;P_{0}\left(0\right)=1,$$

where $B_{0}\left(t\right)$ is the nucleation rate which is a function of states that change with time t (more details below), $P_{0}\left(t\right)$ is the time evolution of the probability that the droplet contains no crystals, and V(t) is the volume of the droplet. The analytical solution of Equation (1) is

(2) $$P_{0}\left(t\right)=e^{-\int_{0}^{t}B_{0}\left(s\right)V\left(s\right)ds}.$$

The induction time $t_{\text{ind}}$ is the time when at least one crystal has nucleated. The cumulative distribution function (CDF) for the induction time and the corresponding probability distribution function (PDF) are

(3) $$F\left(t\right)=1-P_{0}\left(t\right)=1-e^{-\int_{0}^{t}B_{0}\left(s\right)V\left(s\right)ds},$$

(4) $$f\left(t\right)=\frac{\;d\;F\left(t\right)}{\;d\;t}=B_{0}\left(t\right)V\left(t\right)e^{-\int_{0}^{t}B_{0}\left(s\right)V\left(s\right)ds},$$

and the mean induction time is

(5) $$\begin{array}{ccc} {\bar{t}}_{\text{ind}} & = & \int_{0}^{\infty }tf\left(t\right)dt=\int_{0}^{\infty }tdF\left(t\right)=-\int_{0}^{\infty }tdP_{0}\left(t\right)={\left[-tP_{0}\left(t\right)\right]}_{0}^{\infty }+\int_{0}^{\infty }P_{0}\left(t\right)dt\\ & = & \int_{0}^{\infty }P_{0}\left(t\right)dt=\int_{0}^{\infty }e^{-\int_{0}^{t}B_{0}\left(s\right)V\left(s\right)ds}dt. \end{array}$$

The nucleation rate in the droplet is modeled by the classical homogeneous nucleation expression (Nielsen, 1964),

(6) $$B_{0}\left(t\right)V\left(t\right)=AC_{P}\left(t\right)V\left(t\right)\text{exp}(-\frac{B}{{\left(\ln S\left(t\right)\right)}^{2}}),$$

where A and B are nucleation parameters, $S\left(t\right)=C_{P}\left(t\right)∕C_{P,\;\text{sat}\;}\left(t\right)$ is the supersaturation, $C_{P}\left(t\right)$ is the concentration of protein, and $C_{P,\;\text{sat}\;}\left(t\right)$ is the solubility. The most rigorous definition for the supersaturation is in terms of chemical potentials but S(t) is nearly always written in terms of concentrations to avoid the time and expense of computing the chemical potential of the solution phase. Although there is substantial evidence that not all primary nucleation is described by classical nucleation theory (Erdemir et al., 2009), the above expression has been shown to correlate well with experimental data for most solute‐solvent systems while having only two fitting parameters (Kim & Mersmann, 2001).

Since the amount of protein in the droplet $C_{P}\left(t\right)V\left(t\right)$ is constant, the nucleation rate in the droplet (Equation 6) is a monotonically increasing function of supersaturation. Before the first crystal forms, the supersaturation and nucleation rate increase, until the droplet reaches an equilibrium volume with respect to the reservoir solution. Then

(7) $$\int_{0}^{t}B_{0}\left(s\right)V\left(s\right)ds<B_{0,e}V_{e}t,$$

(8) $${\bar{t}}_{\;\text{ind}\;}=\int_{0}^{\infty }e^{-\int_{0}^{t}B_{0}\left(s\right)V\left(s\right)ds}dt>\int_{0}^{\infty }e^{-B_{0,e}V_{e}t}dt=\frac{1}{B_{0,e}V_{e}},$$

where subscript “*e*” refers to the conditions in the droplet at the equilibrium volume before nucleation. This expression can be rearranged to provide a lower bound for the nucleation rate of

(9) $$B_{0,e,\;\text{lb}\;}=\frac{1}{{\bar{t}}_{\;\text{ind}\;}V_{e}},$$

where subscript “lb” indicates a lower bound. Some publications directly report an induction time assuming that the time for a nucleus to grow large enough to be observable is negligible (Scott et al., 2005). Other publications report only the total time for nucleation and growth (Dormitzer et al., 2002; Kraschnefski et al., 2008, 2005; Yu et al., 2008; Zhang et al., 2007). The above lower bound remains valid, although less tight, for the induction times reported in these publications.

Numerous expressions are available for modeling the crystal growth rate G(t), which are all increasing functions of supersaturation. The supersaturation is maximum before nucleation because the supersaturation decreases after nucleation due to the crystal growth. Then the growth rate can be related to the mean crystal size $\bar{L}\left(t\right)$ by

(10) $$\bar{L}\left(t\right)=\frac{1}{N\left(t\right)}\sum_{n=1}^{N\left(t\right)}\int_{t_{n}}^{t}G\left(s\right)ds<\int_{t_{\;\text{ind}\;}}^{t}G\left(s\right)ds<G_{e}\left(t-t_{\;\text{ind}\;}\right),$$

where N(t) is the number of crystals, $t_{n}$ is the time when at least n crystals have nucleated, and the subscript “*e*” refers to the droplet conditions before nucleation. The first inequality is introduced because the times when the crystals have nucleated cannot be directly measured. This expression can be rearranged to provide a lower bound for the crystal growth rate,

(11) $$G_{e,\;\text{lb}\;}=\frac{\bar{L}\left(t\right)}{t-t_{\;\text{ind}\;}}.$$

As before, when publications do not report the induction time directly, this lower bound remains valid while being less tight.

Table 1 reports the lower bounds on the crystal nucleation and growth rates calculated for the literature‐reported crystallization results for truncated VP8 subunit proteins (Dormitzer et al., 2002; Kraschnefski et al., 2008, 2005; Scott et al., 2005; Yu et al., 2008; Zhang et al., 2007). Although the estimated crystallization rates are very slow, evaporation‐based crystallization should be feasible for truncated VP8 subunit proteins by providing a method for controlling supersaturation to deal with the uncertainties in the crystallization kinetics. The slow primary nucleation rate is addressable by seeding, and the slow crystal growth rate is addressable by increasing the surface area of the crystals. These estimated crystallization rates can be applied for the preliminary design of evaporative crystallizers.

**Table 1:** Estimated lower bounds on the crystal nucleation and growth rates for truncated VP8 subunit proteins of rotaviruses

Protein	Temp (°C)	$C_{P,e}$ (g/L)	$B_{0,e,\;\text{lb}\;}$ $\left(10^{-3}{\mathrm{\mu L}}^{-1}h^{-1}\right)$	$G_{e,\;\text{lb}\;}\left(\mathrm{\mu m}/h\right)$	References
NCDV (P[1]) $\text{VP}8_{64-224}^{*}$	30	20	8.33–13.9	2.18–3.64	Yu et al. (2008)
RRV (P[3]) $\text{VP}8_{62-224}^{*}$		17.6	0.99–6.94	0.425–2.97	Dormitzer et al. (2002)
RRV (P[3]) $\text{VP}8_{64-224}^{*}$		40	2.98–6.94	4.85–11.3	Kraschnefski et al. (2008)
CRW‐8 (P[7]) $\text{VP}8_{64-224}^{*}$	20	20	20.8	0.128	Scott et al. (2005)
OSU (P[7]) $\text{VP}8_{65-224}^{*}$		30	2.98	1.07	Zhang et al. (2007)
		10	20.8	2.22	
Wa (P[8]) $\text{VP}8_{64-223}^{*}$		30	4.17	1.05	Kraschnefski et al. (2005)
		20	0.372	0.0700	

### 2.2 Identification of feasible crystallization conditions

**Figure 1:**
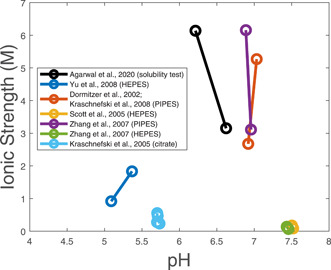
Crystallization and solubility test conditions for truncated VP8 subunit proteins [Color figure can be viewed at wileyonlinelibrary.com]

To identify feasible crystallization conditions for truncated VP8 subunit proteins, a mechanistic model developed for predicting the pH and ionic strength of cell culture media (Hong et al., 2021) was applied to the literature‐reported crystallization media (Dormitzer et al., 2002; Kraschnefski et al., 2008, 2005; Scott et al., 2005; Yu et al., 2008; Zhang et al., 2007) (Table 2). All of the studies in the literature mixed equal volumes of the sample solution containing truncated VP8 subunit protein and the reservoir solution containing buffers and precipitants. The various types of buffers and precipitants in the reservoir solutions resulted in a wide range of values for pH and ionic strength in which crystallization occured (Figure 1). These results show that crystallization of truncated VP8 subunit proteins is feasible under a wide range of conditions.

**Table 2:** Crystallization media for truncated VP8 subunit proteins of rotaviruses (TNE: 20 mM Tris‐HCl pH 8.0, 100 mM NaCl, 1 mM EDTA)

Sample solution	Reservoir solution	References
TNE	1.6 M $\text{Na}/{\text{KPO}}_{4}$, 0.1 M HEPES pH 7.5	Yu et al. (2008)
5.6 mM Tris‐HCl pH 8.0, 14 mM ${\text{NaPO}}_{4}$ pH 7.0, 35 mM NaCl, 0.3 mM EDTA, 0.02% ${\text{NaN}}_{3}$, 0.1 mM benzamidine	1.7 M ${\left({\text{NH}}_{4}\right)}_{2}{\text{SO}}_{4}$, 2.4% (v/v) PEG 400, 0.1 M PIPES pH 6.5	Dormitzer et al. (2002), Kraschnefski et al. (2008)
TNE	70% 2‐methyl‐2,4‐pentanediol, 0.1 M HEPES pH 7.5	Scott et al. (2005)
6 mM Tris‐HCl pH 8.0, 16 mM NaPO4 pH 7.0, 35 mM NaCl, 0.3 mM EDTA	2 M ${\left({\text{NH}}_{4}\right)}_{2}{\text{SO}}_{4}$, 3% PEG 400, 0.1 M PIPES pH 6.5	Zhang et al. (2007)
	70% 2‐methyl‐2,4‐pentanediol, 0.1 M HEPES pH 7.5	
TNE	25% (w/v) PEG 4000, 0.1 M sodium citrate pH 5.6, 20% (v/v) 2‐propanol	Kraschnefski et al. (2005)
	11.7% (w/v) PEG 4000, 0.08 M sodium citrate pH 5.6, 16% (v/v) 2‐propanol, 19% (v/v) ethylene glycol	

### 2.3 Proof‐of‐concept crystallization experiments

After the above analyses of the literature were carried out, crystallization experiments were performed for a modified P2‐VP8‐P[8] with reduced aggregation and glycosylation. Solubility tests for NRRV antigens in 10 mM PBS buffer (pH 7.2) indicate that the protein solubility significantly decreases with ammonium sulfate from about 1 to 2 M (Agarwal et al., 2020). The crystallization medium with a starting concentration of 1 M ammonium sulfate was chosen with PIPES buffer (pH 6.5) so as to have similar pH and ionic strength as in past crystallization studies (Figure 1).

**Figure 2:**
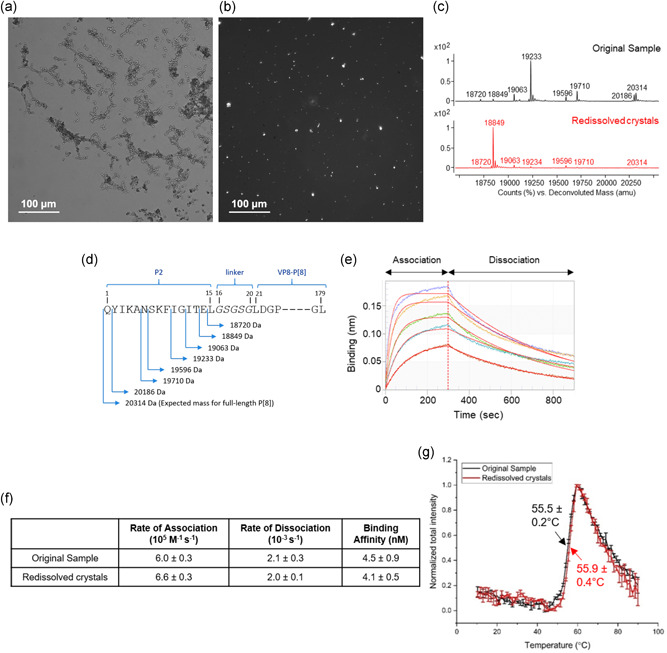
(a) In situ and (b) cross‐polarized microscope images of P2‐VP8‐P[8] crystals. (c) Intact protein mass analysis and (d) N‐terminal amino acid sequence indicating various mass species (P2 epitope truncated variants) observed in the mass spectra. (e) Representative sensograms for original sample and mAb interaction, and (f) binding and kinetic parameters measured using Bio‐layer Interferometry of both samples (n=3, 1 *SD*). (g) Extrinsic fluorescence spectroscopy versus temperature indicating mean thermal melting temperature ($T_{m}$) and 1 *SD* from triplicate analysis [Color figure can be viewed at wileyonlinelibrary.com]

Crystallization was first performed using the hanging‐drop vapor diffusion system. The in situ microscope image in Figure 2a shows the formulation of P2‐VP8‐P[8] particles. These particles were observed to be white when placed on a glass slide and imaged in a cross‐polarized light microscope (Figure 2b), which indicates that the P2‐VP8‐P[8] particles are anisotropic and in the crystalline state. This proof‐of‐concept experiment showed that controlled evaporation with the feasible crystallization conditions identified from the literature resulted in the nucleation and growth of P2‐VP8‐P[8] crystals.

Scaled‐up evaporative crystallization, in the order of 100 μl, was performed to create samples for analytical characterization. Intact protein mass analysis of the protein samples before and after crystallization showed the presence of full‐length and truncated variants (truncations localized in the P2 epitope region) in both the original and redissolved crystal samples (Figure 2c,d). The abundance of these observed species, however, varied between the two samples that may be a result of preferential crystallization of the smaller truncated species, where less of the flexible N terminus region is better able to crystallize. Additionally, when comparing the original and redissolved crystal samples, no significant differences were observed in the ability of the NRRV antigen's antibody binding profile (Figure 2e,f) or the NRRV antigen's overall conformational stability (Figure 2g). These results demonstrate the promise of evaporative crystallization of the NRRV antigen.

## 3 MATERIALS AND METHODS

### 3.1 Fermentation and purification

The P2‐VP8‐P[8] sequence was modified to improve product quality and expression titer (Dalvie et al., 2020). The modified P2‐VP8‐P[8] was expressed and secreted from *Pichia pastoris* (*Komagataella phaffii* NRRL Y‐11430). The fermentation and protein purification were carried out in an automated, benchtop, multiproduct manufacturing system, as previously reported (Crowell et al., 2018). Cells were grown with 4% glycerol for biomass accumulation and 1% methanol, supplemented with 67 g/L sorbitol, for production. The temperature, pH, and dissolved oxygen were maintained at 25°C, 6.5, and 25%, respectively.

Purified protein was concentrated approximately 10‐fold using 3.5 kDa molecular weight cut off (MWCO) Amicon Ultra Centrifugal Filter Units (Millipore Sigma) according to the manufacturer's recommended protocol. The concentrated protein was then dialyzed against 0.1 M PIPES, pH 6.5, using a 3.5 kDa MWCO Slide‐A‐Lyzer G2 dialysis cassette (Thermo Fisher Scientific) according to the manufacturer's recommended protocol.

### 3.2 Crystallization experiments

Crystallization was performed using a hanging‐drop vapor diffusion system (Hampton Research, VDX Plate) with 5 µl droplet of a sample solution containing 8.5 g/L P2‐VP8‐P[8] and 0.1 M PIPES (pH 6.5) mixed with an equal volume of a reservoir solution containing 2 M ammonium sulfate. In situ microscope images were taken using a microscope (Leica, Model DM2500) with a camera (Sony, Model ILCE‐5100). After crystallization is finished from the hanging‐drop vapor diffusion system, the glass cover slide containing the droplet was transferred to an air‐dusted glass microscope slide to take cross‐polarized images.

Scaled‐up evaporative crystallization was performed with 200 µl of the sample solution mixed with an equal volume of the reservoir solution. The solution was evaporated with average rate of 1.6 mg/h until the final volume is halved. Then the crystals in the solution were filtered (Millipore, Membrane Filter, 0.22 µm pore size), washed, and redissolved in 0.1 M PIPES (pH 6.5).

### 3.3 Analytical characterization

Intact protein mass analysis was performed using a time‐of‐flight LC/MS system (Agilent Technologies, 6230B) with a HPLC system (Agilent Technologies, 1220). About 15 to 20 pmol of each sample was injected and desalted using a ZORBAX column (Agilent Technologies, 300SB‐C3). The LC gradient consisted of 20% to 70% B (A: water with 0.1% formic acid, B: acetonitrile with 0.1% formic acid) over 1 min at 1.5 ml/min. Protein elution was monitored using the absorbance signal at 214 nm. A volume of 100 µl of isopropanol was injected after each sample to control sample carry‐over. The typical electrospray ionization parameters were $290^{\circ }$C gas temperature, 4000 V Vcap, and 275 V fragmentor. Mass spectra were collected from 700 to 2800 *m*/*z* at 1 spectra/second and processed using MassHunter Qualitative Analysis (Agilent Technologies) with deconvolution range of 10–50 kDa and 1 Da mass step.

The antibody binding test was studied using an Octet RED96 (Bio‐layer Interferometry) system with high‐precision streptavidin biosensors (Pall ForteBio). P[8]‐specific monoclonal antibody was biotinylated using the EZ‐Link Sulfo‐NHS‐LC‐biotinylation kit (Thermo Fisher Scientific) and used in the assay at optimized loading concentrations of 1 µg/ml. Protein samples were loaded in a 96‐well black microplate (Greiner Bio‐One) at optimized starting concentrations of 5 µg/ml and serially diluted by six twofold dilutions. Association and disassociation were measured for 300 and 600 s at the shake speed of 1000 rpm. Binding affinity was calculated using the Octet Data Analysis (Pall ForteBio) with Savitzky‐Golay filtering, 1:1 binding model, and global fitting.

Extrinsic fluorescence spectroscopy was performed using a fluorescence plate reader (Fluorescence Innovations) (Wei et al., 2018). 8‐Anilino‐1‐naphthalene sulfonate (ANS) was used as the extrinsic fluorescence probe. Samples were prepared using dye to protein molar ratio of 25:1 and excited at 350 nm using a combo laser. The time‐resolved fluorescence (TRF) was measured using a 405 nm long‐pass dichroic mirror, a band‐pass filter (485±20 nm), and a photomultiplier tube (PMT). Measurements were performed using integration time of 500 ms and PTM voltage of 500 V. Samples were scanned using a 10°C–$90^{\circ }$C temperature ramp at a rate of 1.25°C/min. The TRF mode measures fluorescence decay waveforms (Wei et al., 2018). Total intensity data (the peak area under the curve for a waveform) at various temperatures were acquired from the plate reader and normalized using min–max normalization. Origin 9.4 software package was used to calculate the $T_{m}$ value by plotting first derivative of total intensity data against corresponding temperatures.

## AUTHOR CONTRIBUTIONS

*Conceptualization*: Moo Sun Hong, Richard D. Braatz; *Modeling*: Moo Sun Hong; *Crystallization Experiments*: Moo Sun Hong; *Analytical Characterization*: Moo Sun Hong, Kawaljit Kaur, Nishant Sawant, Sangeeta B. Joshi, and David B. Volkin; *Writing—original draft*: Moo Sun Hong, Kawaljit Kaur, Nishant Sawant, Sangeeta B. Joshi, and David B. Volkin; *Writing—review and editing*: Moo Sun Hong, Richard D. Braatz; Funding Acquisition: Richard D. Braatz, and David B. Volkin.
